# Comparison of the Efficacy of Filgrastim and an Inactivated *Parapoxvirus ovis* Paraimmune Activator in Naturally Infected Cats with Feline Panleukopenia

**DOI:** 10.3390/ani16071066

**Published:** 2026-03-31

**Authors:** Emre Tüfekçi, Gencay Ekinci, Serkan Kökkaya, Muhammed Arif Toy, Alfatih Mohammed Ahmed Abozaid, Ekrem Gülcek, Rabia Tüfekçi, Vehbi Güneş, Mehmet Çitil, İhsan Keleş

**Affiliations:** 1 Department of Wild Animal Diseases, Faculty of Veterinary Medicine, Erciyes University, 38000 Kayseri, Türkiye; 2 Department of Internal Medicine, Faculty of Veterinary Medicine, Erciyes University, 38000 Kayseri, Türkiye; gekinci@erciyes.edu.tr (G.E.); vgunes@erciyes.edu.tr (V.G.); mehmetcitil@erciyes.edu.tr (M.Ç.); ihsankeles@yahoo.com (İ.K.); 3 Department of Virology, Faculty of Veterinary Medicine, Yozgat Bozok University, 66000 Yozgat, Türkiye; serkan.kokkaya@yobu.edu.tr; 4 Department of Veterinary Microbiology, Institute of Health Sciences, Erciyes University, 38000 Kayseri, Türkiye; mariftoyy@gmail.com; 5 Department of Veterinary Internal Medicine, Institute of Health Sciences, Erciyes University, 38000 Kayseri, Türkiye; fatihmohammed1995@gmail.com (A.M.A.A.); ekremgulcek.eg@gmail.com (E.G.); 6 Department of Veterinary Physiology, Institute of Health Sciences, Selçuk University, 42100 Konya, Türkiye; 203144001001@ogr.selcuk.edu.tr

**Keywords:** feline, feline panleukopenia, feline panleukopenia virus, filgrastim, hG-CSF inactivated *Parapoxvirus ovis*, paraimmune activator

## Abstract

Feline panleukopenia (FPL) is a serious viral disease caused by *Feline panleukopenia virus* (FPV) that causes leukopenia, lymphopenia, and neutropenia, particularly in young or unvaccinated cats. There is no specific antiviral treatment available for FPL, and treatment protocols generally consist of fluid therapy and supportive care. This study evaluated the clinical and hematological efficacy of filgrastim, a granulocyte colony-stimulating factor (G-CSF) that has shown successful results in treating FPL in various studies, and the paraimmune activator-inactivated *Parapoxvirus ovis* (iPPVO) in 49 cats naturally infected with FPV. Cats were randomly assigned to four groups: low-dose filgrastim (5 µg/kg, n = 13), high-dose filgrastim (20 µg/kg, n = 14), iPPVO (n = 12), and standard supportive treatment (n = 10). Clinical signs and complete blood counts were assessed on days 0 and 7. By day 7, high-dose filgrastim showed greater increases in white blood cell, lymphocyte, monocyte, and neutrophil counts compared with the other groups (*p* < 0.05), whereas moderate improvements were observed in the iPPVO group. Leukopenia and lymphopenia resolved faster in the high-dose filgrastim group than in the low-dose filgrastim and standard treatment groups. Clinical recovery, including reduction in vomiting and lethargy, was more pronounced in the high-dose filgrastim and iPPVO groups. Survival rates did not differ significantly among groups (*p* = 0.615), although the high-dose filgrastim group showed the lowest mortality (42.9%). These findings suggest that high-dose filgrastim may contribute to cytopenias and promote hematological recovery in FPL, while iPPVO may serve as a supportive immunomodulatory therapy. However, it should be noted that the efficacy of filgrastim and/or iPPVO treatments has not been definitively confirmed, likely due to the small sample size and the lack of well-controlled randomized studies.

## Simple Summary

Feline panleukopenia is a serious viral disease that affects domestic cats and can be life-threatening, particularly in young and unvaccinated animals. The disease severely suppresses the bone marrow, leading to reduced numbers of white blood cells, lymphocytes, and neutrophils, which weakens the cat’s immune system and increases the risk of secondary infections. This study compared the effects of high- and low-dose filgrastim, a hematopoietic agent that stimulates leukocyte production, with an immunomodulatory paraimmune activator, inactivated *Parapoxvirus ovis* (iPPVO), in cats naturally infected with the virus. Clinical signs such as vomiting, diarrhea, lethargy, and anorexia, as well as blood cell counts, were monitored over a week. The findings show that high-dose filgrastim led to better improvement in blood counts compared with the other treatments. iPPVO provided moderate benefits, while standard treatment alone was less effective. These results suggest that high-dose filgrastim can improve the hematological parameters recovery in infected cats and may help reduce the severity of disease. However, it should be noted that the efficacy of filgrastim and/or iPPVO treatments has not been definitively confirmed, likely due to the small sample size and the lack of well-controlled randomized studies.

## 1. Introduction

Feline panleukopenia (also called feline distemper) is a highly contagious and life-threatening viral pathogen of domestic and wild cats [1]. The etiological agent, called *Feline panleukopenia virus* (FPV) for many years, was renamed *Protoparvovirus carnivoran 1* in 2022 [2]. The virus belongs to the family *Parvoviridae*, genus *Protoparvovirus*, and it is closely related to *Canine parvovirus* [3]. FPV is characterized by its remarkable environmental resistance and its ability to persist for long periods under adverse conditions, which contributes significantly to its widespread distribution and high transmission rates among susceptible feline populations [4,5].

Natural infection with FPV occurs primarily via the fecal-oral route through direct contact with infected animals or contaminated environments. Kittens and unvaccinated young cats are particularly susceptible, and infection is often associated with high morbidity and mortality [1,6]. Following viral entry, FPV preferentially targets rapidly dividing cells, especially those of the intestinal crypts, bone marrow, and lymphoid tissues [7,8]. This tropism results in severe gastrointestinal damage, immunosuppression, and profound leukopenia, which are hallmarks of the disease [5].

Clinically, feline panleukopenia (FPL) manifests with nonspecific signs such as depression, lethargy, dehydration, anorexia, fever, vomiting, diarrhea (watery or hemorrhagic), and rapid deterioration of the general condition [3,4,9,10]. One of the most critical pathological features of FPV infection is bone marrow suppression leading to panleukopenia, which predisposes affected cats to secondary bacterial infections and worsens the prognosis [10]. Therefore, hematological evaluation, particularly leukocyte counts, plays a crucial role in both diagnosis and monitoring of disease progression [11].

Currently, there is no specific antiviral therapy available for FPL. Treatment is mainly supportive and focuses on fluid therapy, correction of electrolyte imbalances, control of secondary infections, and nutritional support [12,13]. Immunomodulatory therapies, which aim to enhance the host immune response and improve survival rates in affected cats, have recently garnered increasing interest [3].

Filgrastim is a recombinant human granulocyte colony-stimulating factor (rHuG-CSF) that promotes neutrophil production and accelerates recovery from leukopenia [14,15]. In human medicine, filgrastim is widely used to prevent chemotherapy-induced neutropenia and to mobilize stem cells prior to transplantation [16,17]. In veterinary medicine, filgrastim has been used to stimulate bone marrow activity and shorten the duration of neutropenia, thereby reducing the risk of secondary infections in cats and dogs infected with parvovirus [18,19,20]. Besides parvoviral infections, its use has also been reported in dogs and cats with chemotherapy-induced neutropenia, bone marrow suppression, and severe infectious diseases [15,21]. Studies in dogs have also shown that human G-CSF can enhance neutrophil release from the bone marrow [21,22,23]. Filgrastim administration was associated with a significant increase in white blood cell (WBC), lymphocyte, and granulocyte counts between days 0 and 7 in FPV-infected cats, indicating improved hematological recovery, while no adverse effects on serum biochemical parameters were observed [24].

Another immunomodulatory approach involves the use of paraimmune activators derived from inactivated *Parapoxvirus ovis* (iPPVO) [25]. These agents stimulate innate immune responses by activating macrophages and natural killer cells and inducing cytokine production [26,27,28,29]. In veterinary medicine, *Parapoxvirus ovis*-based paraimmune activators have been widely used as supportive treatment in various viral and bacterial infections to enhance nonspecific immunity [29,30,31]. iPPVO is a lyophilized injectable formulation that stimulates the nonspecific immune system and contributes to the prevention of infection- and stress-related diseases in cats, dogs, horses, cattle, and pigs [32,33,34].

Although the individual clinical benefits of filgrastim (at various doses) and iPPVO have been documented [3,24], there are no studies in the veterinary literature directly comparing these two immunomodulatory strategies in naturally occurring FPL. Inconsistencies among previous studies hinder definitive conclusions regarding relative clinical efficacy or the superiority of one agent over the other. Furthermore, a significant knowledge gap remains concerning the dose-dependent response of filgrastim in the context of acute viral bone marrow suppression. Without direct comparative data, clinicians lack an evidence-based framework for determining the optimal immunomodulatory intervention. Therefore, there is a critical need for prospective studies evaluating the comparative outcomes of filgrastim and iPPVO. Addressing these limitations is essential to improve treatment protocols and reduce morbidity and mortality in cats affected by FPL. Therefore, the present study aimed to compare the clinical and hematological efficacy of filgrastim and iPPVO in cats naturally infected with FPV. By evaluating both clinical recovery and changes in hematological parameters, this study sought to provide evidence-based insights into the relative effectiveness of these two therapeutic approaches in naturally occurring infections.

## 2. Materials and Methods

### 2.1. Ethical Approval

The study protocol was approved by the Erciyes University Animal Experiments Local Ethics Committee (approval no: 25/198). All owners provided informed consent before their animals were included in the study.

### 2.2. Study Design

Single-centre prospective study.

### 2.3. Animals

This study included a total of 49 client-owned cats naturally infected with FPV that were presented to Erciyes University, Faculty of Veterinary Medicine, Veterinary Teaching Hospital (VTH) between October 2023 and February 2025. The median age of the cats was 6 months (range: 2–13 months). Of the 49 cats, 26 were male, and 23 were female. Breed distribution consisted of 21 domestic shorthair (tabby) cats, 16 British Shorthair, 9 Scottish Fold, 1 Van cat, and 2 mixed-breed cats.

### 2.4. Grouping of Animals

Clinical assessment and treatment allocation were performed by a single investigator. Allocation was guided by hematological parameters (e.g., leukocyte counts) and clinical severity (vomiting, diarrhea, lethargy, anorexia). Following diagnosis, cats were randomly assigned to four treatment groups, with each cat allocated to a specific group according to the therapeutic protocol. The study reflects a prospective clinical investigation conducted under routine veterinary conditions, with randomization performed at the time of enrolment using a simple random approach.

Low-dose filgrastim (LDF) group, n = 13: Cats treated with filgrastim at a dose of 5 µg/kg.

High-dose filgrastim (HDF) group, n = 14: Cats treated with filgrastim at a dose of 20 µg/kg.

Inactivated *Parapoxvirus ovis* (iPPVO) group, n = 12: Cats treated with inactivated *Parapoxvirus ovis* (iPPVO) as a paraimmune activator.

Standard supportive treatment (ST) group, n = 10: Cats treated with standard supportive therapy alone.

All cats were hospitalized in isolation units to prevent the spread of infection. During hospitalization, each cat was housed in an individual cage; cages were disinfected daily, and personal protective equipment was used during all care procedures. Continuous clinical monitoring was provided for all cases, and isolation and hygiene protocols were strictly enforced. Peripheral venous catheters were placed to allow intravenous fluid therapy, and catheter care was performed on a daily basis. Feeding, medication administration, and clinical examinations were conducted at regular intervals, and all procedures were carried out in accordance with standardized protocols by the same veterinary medical team.

### 2.5. Inclusion and Exclusion Criteria

Cats suspected of FPV infection based on clinical findings (vomiting, diarrhea, lethargy, anorexia, dehydration) and hematological abnormalities (leukopenia) were initially screened using a rapid antigen test. Samples that tested positive by the rapid antigen test were subsequently confirmed by PCR analysis. Only cats with PCR-confirmed FPV infection were included in the study.

Cats that tested positive by the rapid antigen test but were not confirmed by PCR were excluded from the study. In addition, cats were excluded if they had concurrent systemic diseases unrelated to FPL, a prior treatment history, an unknown prognosis, or missing laboratory data (complete blood cell results).

### 2.6. Diagnosis of Feline Panleukopenia

The diagnosis of FPL was established based on a combined evaluation of clinical findings, laboratory diagnostic methods, and hematological assessments. Clinical diagnosis was made according to the inclusion criteria. For laboratory diagnosis, a rapid antigen test was performed on fecal samples, and PCR analysis was used for confirmation. In addition, hematological analyses were conducted in all cats to assess the presence and severity of leukopenia; in particular, a decrease in total leukocyte count below the reference range was considered a supportive diagnostic finding. Cases to be included in the study were determined through the integrated evaluation of clinical and laboratory findings.

### 2.7. Feline Panleukopenia Virus Antigen Detection Using a Rapid Diagnostic Kit

Based on clinical examination findings, a rapid diagnostic test kit was used for antigen detection in suspected cases of FPL. In this context, FPV antigen in fecal samples was analyzed using a lateral flow immunochromatographic (LFI) rapid test kit (Felivet FPV, Hangzhou Evegen Biotech Co., Ltd., Hangzhou, Zhejiang, China). Prior to analysis, all materials included in the test kit were brought to room temperature, and the test device was placed on a flat surface. Rectal fecal samples were collected from the cats using sterile swabs. The collected swab samples were immersed in a tube containing sample buffer and mixed for 10 s to ensure homogenization. The tube was then placed on a flat surface and allowed to stand for 3 min. Following sedimentation of particulate matter at the bottom of the tube, the supernatant was aspirated, and three drops of the sample were dispensed into the sample well. Test results were read within 5 min. The appearance of a single line in the observation window was interpreted as a negative result, whereas the presence of two lines was considered positive.

### 2.8. PCR Analysis

Cats were initially screened using a rapid FPV antigen test. Fecal samples that tested positive with the rapid diagnostic kit were subsequently analyzed by PCR for confirmation of FPV infection, and only PCR-positive cases were included in the study. For this purpose, DNA was extracted from the samples using the Nucleogene Viral DNA Extraction Kit (REF: NGE003, Nucleogene, Istanbul, Türkiye) in accordance with the manufacturer’s instructions. PCR amplification was performed using Ampliqon Taq DNA Polymerase (Cat. No: A111103, Ampliqon, Odense, Denmark). The PCR master mix was prepared to a final volume of 50 µL and consisted of 1 µL forward primer (5′-CATACATGGCAAACAAATAGAGCA-3′), 1 µL reverse primer (5′-TGTTTTAAATGGCCCTTGTGTAGA-3′) [8], 5 µL DNA template, 1 µL 10 mM dNTP mix, 5 µL 10× buffer, 0.5 µL Taq DNA polymerase, and 36.5 µL nuclease-free water (Sigma-Aldrich, W4502, Steinheim, Germany). PCR amplification was carried out in a thermal cycler (Bio-Rad, T100, Hercules, CA, USA) under the following conditions: initial denaturation at 95 °C for 5 min, followed by 35 cycles of denaturation at 95 °C for 30 s, annealing at 53 °C for 30 s, and extension at 72 °C for 1 min, with a final extension step at 72 °C for 10 min. In each PCR run, a previously confirmed FPV-positive sample from the laboratory was included as a positive control, while nuclease-free water served as the negative control. PCR amplicons were separated by electrophoresis on a 1.5% agarose gel containing ethidium bromide and visualized using a UV transilluminator (Vilber Lourmat, Collégien, France).

### 2.9. Treatment Protocols

#### 2.9.1. Filgrastim Treatment Protocol

As part of the treatment protocol, filgrastim (recombinant methionyl human granulocyte colony-stimulating factor [r-metHuG-CSF]) (Neupogen^®^, syringe containing 30 million units/0.5 mL, corresponding to 300 μg of filgrastim in 0.5 mL; Amgen Inc., Thousand Oaks, CA, USA) was administered subcutaneously (SC) at doses of 5 µg/kg and 20 µg/kg, once daily for five consecutive days. The filgrastim treatment protocol was implemented in accordance with the protocol reported by Morris and Dobson [35].

#### 2.9.2. Inactivated *Parapoxvirus ovis* Paraimmune Activator Protocol

Within this protocol, twelve cats received the iPPVO E(P)CK strain (Rapidoll^®^, Dollvet Biotechnology Inc., İstanbul, Türkiye) administered subcutaneously (SC) at a dose of 1 mL per cat (minimum 256 interferon units), given at 48-h intervals for a total of three doses.

#### 2.9.3. Supportive Treatment

Supportive treatment was applied uniformly across all groups, and all cases received standard supportive care for a duration of 7 days. As part of the supportive therapy, crystalloid fluid therapy was administered using isotonic saline at a dose of 44 mL/kg once daily via the intravenous (IV) route (Polifeks^®^, Polifarma, Ergene, Türkiye). Antimicrobial therapy was initiated immediately in all cats, primarily targeting Gram-negative bacteria, particularly *Escherichia coli*. For this purpose, enrofloxacin (Baytril K 5%^®^, Bayer, Leverkusen, Germany) was administered subcutaneously (SC) at a dose of 5 mg/kg once daily. All cats received maropitant citrate (Cerenia^®^, Zoetis, Parsippany, NJ, USA) as an antiemetic and famotidine (Pepsid^®^, Merck & Co., Inc., Rahway, NJ, USA) for gastric protection as part of the supportive treatment protocol. In addition, ascorbic acid (Maxivit-C^®^, Bavet, Istanbul, Türkiye) was administered intramuscularly (IM) at a dose of 30 mg/kg once daily as vitamin supplementation. Vitamin B12 (Berovit B12^®^, Ceva, Istanbul, Türkiye) supplementation was also provided at doses of 0.05–0.1 mL in kittens and 0.25 mL in adult cats, administered daily or every other day.

Nutritional support was planned to avoid triggering vomiting and to prevent the development of hepatic lipidosis. Accordingly, high-energy, easily digestible diets (Hill’s a/d^®^) were offered in small, frequent meals. In cases unable to tolerate oral feeding, energy gel preparations were applied to the gingiva, or supportive care was maintained solely with subcutaneous fluid therapy combined with parenteral glucose supplementation (Polifeks^®^ Dextrose %5, Polifarma, Ergene, Türkiye).

### 2.10. Clinical Evaluation

The cats included in the study were regularly monitored on days 0 and 7 for various clinical parameters, including overall clinical condition, such as appetite, fever, diarrhea, vomiting, and lethargy. The presence of fever was assessed through body temperature measurements, while gastrointestinal signs were documented by recording the occurrence of diarrhea and vomiting. In addition, the general clinical status of the animals was evaluated by considering activity level, appetite, and overall behavior.

#### 2.10.1. Blood Collection

Blood samples were collected from the cephalic vein (*vena cephalica antebrachii*) of each cat into BD Vacutainer^®^ tubes (Becton Dickinson, Franklin Lakes, NJ, USA) on day 0 (before initiation of treatment) and on day 7.

#### 2.10.2. Hematological Analysis

Complete blood count (CBC) analyses were performed using an automated hematology analyzer (Exigo Eos^®^, Boule Diagnostics, Spånga, Sweden). The evaluated hematological parameters included total WBC counts, leukocyte differential counts, red blood cell (RBC) count, and platelet (Plt) count.

### 2.11. Outcome Measures

The primary outcome measures of the study were defined as the increase in leukocyte count following treatment and the survival rate.

### 2.12. Statistical Analysis

Statistical analyses were performed using commercial software (SPSS for Windows version 25.0, SPSS Inc., Chicago, IL, USA). All data were first visually inspected, and the Shapiro–Wilk test was used to assess the normality of the distribution. Descriptive statistics were then summarized. Data that met the assumption of normality were expressed as means ± standard deviation. Variables that did not pass the normality test are reported as median (min-max). To account for baseline differences, day 7 hematological parameters were analyzed using analysis of covariance (ANCOVA), with treatment group as the fixed factor and the corresponding day 0 value included as a covariate. Bonferroni and Tukey’s honestly significant difference (HSD) tests were applied for post hoc comparisons. For comparisons of paired measurements obtained from the same individuals at day 0 (baseline) and day 7, the paired samples *t*-test was used for normally distributed data, whereas the Wilcoxon signed-rank test was employed for non-normally distributed variables. Categorical variables were analyzed using Fisher’s exact test, whereas analysis of age differences between groups was performed using ANOVA. A *p*-value less than 0.05 was considered statistically significant in all analyses.

## 3. Results

### 3.1. Clinical Findings

When the age distributions among the groups were compared, the median age values were determined as 12 months (5.5–12) in the LDF group, 5.5 months (2.75–8.25) in the HDF group, 5.5 months (4–7.5) in the iPPVO group, and 12 months (4.75–18) in the ST group. Statistical analysis revealed no significant difference in age among the groups (*p* = 0.611) (Table 1).

No statistically significant difference was detected among the groups in terms of sex distribution (Fisher’s exact test; χ^2^ = 1.809, *p* = 0.656). This finding indicates that the groups were homogeneous with respect to sex and that the treatment groups were comparable in terms of sex distribution (Table 1).

When the breed distributions of the cats included in the study were compared among the groups, statistically significant differences were identified (Fisher’s exact test; χ^2^ = 15.200, *p* = 0.095). British Shorthair cats were most frequently represented in the LDF group (53.8%; 7/13), whereas lower proportions were observed in the HDF (28.6%; 4/14), iPPVO (33.3%; 4/12), and ST (10%; 1/10) groups. Scottish Fold cats were more commonly observed, particularly in the LDF (30.8%; 4/13) and ST (30%; 3/10) groups, while only a limited number were present in the HDF and iPPVO groups. Tabby cats were the predominant breed in the HDF (64.3%; 9/14), iPPVO (50%; 6/12), and ST (60%; 6/10) groups, whereas no tabby cats were present in the LDF group. Mixed-breed cats were identified only in the LDF group (15.4%; 2/13), while Van cats were represented exclusively in the iPPVO group (8.3%; 1/12) (Table 1).

Clinical findings and body temperature values were evaluated on days 0 and 7 of the study. At baseline (day 0), a high frequency of clinical signs was observed across all groups. On the day 7 assessment, a reduction in the prevalence of clinical signs was noted in all groups. However, this decrease was more pronounced in the filgrastim-treated groups (LDF and HDF) and in the iPPVO group compared with the ST group. Notably, vomiting had completely resolved in the HDF and iPPVO groups, whereas it persisted in 30% (3/10) of the cases in the ST group. Similarly, diarrhea, anorexia, and lethargy were found to persist at higher rates in the ST group on day 7. With regard to body temperature, a statistically significant increase on day 7 was observed in the HDF group (*p* = 0.019), whereas changes in body temperature in the other groups were not statistically significant (*p* > 0.05). Overall, these findings indicate that filgrastim (particularly at a high dose) and iPPVO administration accelerated clinical recovery, while clinical symptoms persisted for a longer duration in the standard treatment group (Table 2).

The fecal samples that were LFI-positive and had compatible clinical findings were then subjected to PCR. The samples were amplified using specific primers for PCR.

A statistically significant difference was identified among the groups with respect to vaccination status (Fisher’s exact test; χ^2^ = 21.993, *p* < 0.001). In the HDF group, vaccinated and unvaccinated cats were equally represented (50–50%), whereas in the iPPVO group, the vast majority of cats were unvaccinated (91.7%; 11/12). In the LDF group, vaccination status was unknown for most cases (69.2%; 9/13). In the ST group, vaccinated and unvaccinated cats were equally distributed (40–40%), and vaccination status was unknown in 20% of the cases (Table 1).

### 3.2. Complete Blood Count Findings

Marked differences were observed among the groups and over time with respect to hemogram parameters (Table 3 and Table 4). Baseline-adjusted analyses using ANCOVA confirmed significant treatment effects for WBC and granulocyte counts, while erythrocyte parameters were primarily influenced by baseline values rather than treatment group. WBC counts at day 7 differed significantly among treatment groups (*p* = 0.001). Post hoc comparisons indicated that cats receiving high-dose filgrastim exhibited significantly higher WBC counts compared with the control group. Similarly, granulocyte counts were significantly affected by treatment (*p* < 0.001), with the greatest increase observed in the high-dose filgrastim group. Monocyte counts showed a modest but statistically significant treatment effect (*p* = 0.047). Lymphocyte counts were also higher in the high-dose filgrastim group compared with the control group, although the overall model approached but did not strongly reach statistical significance (*p* = 0.063) (Table 3).

For erythrocyte-related parameters, including RBC, hemoglobin (Hgb), and hematocrit (Hct), ANCOVA analysis demonstrated that baseline (day 0) values were significant predictors of day 7 measurements (*p* < 0.001 for the covariate effect). However, after adjustment for baseline differences, no statistically significant treatment (group) effects were observed for these variables (Table 4).

Overall, the increases in WBC, lymphocyte, monocyte, and neutrophil counts were more pronounced in the filgrastim-treated groups, whereas moderate increases were observed in the iPPVO group and only limited changes were detected in the standard treatment group. Administration of high-dose filgrastim was associated with increases in WBC, lymphocyte, monocyte, and neutrophil counts, while the LDF and ST groups showed comparatively smaller changes (Table 3).

At baseline (day 0), cytopenias such as leukopenia, lymphopenia, and neutropenia were commonly observed in most groups. In terms of WBC counts, the majority of cases in the LDF and HDF groups were leukopenic (12 and 14 cases, respectively), whereas the number of leukopenic cases was lower in the ST group (6 cases). By day 7, WBC counts in the filgrastim-treated groups (LDF and HDF) showed a tendency toward hematological improvement. Notably, the number of leukopenic cases in the HDF group decreased to three, suggesting a faster recovery of the immune response. In contrast, such improvement was less evident in the iPPVO and ST groups.

A similar trend was observed for lymphocyte counts. At baseline, lymphopenia was prevalent in the LDF and HDF groups (10 and 13 cases, respectively); by day 7, the number of lymphopenic cases in the HDF group had decreased to 4, while the number of cases within the normal range increased to 10. Improvement was also observed in the LDF group, although it was less pronounced than in the HDF group. In the iPPVO and ST groups, improvement in lymphocyte counts remained more limited.

While monocyte and neutrophil counts were more evenly distributed among the groups at baseline, a marked increase was observed in the HDF group by day 7, accompanied by a reduction in the number of cytopenic cases. Granulocyte counts showed particularly rapid improvement in the HDF group. No significant changes were observed in eosinophil percentages either among the groups or over time.

The number of cases presenting with mild anemia or decreased RBC, Hgb, and Hct values at baseline was similar among the groups, and no marked differences were observed on day 7. With regard to Plt counts, the number of thrombocytopenic cases decreased in the filgrastim-treated LDF and HDF groups, accompanied by an increase in the number of cases within the normal range.

Overall, more rapid and pronounced improvement in WBC, lymphocyte, monocyte, and neutrophil counts was observed in the HDF group, whereas the LDF group showed moderate improvement and the iPPVO and ST groups exhibited more limited changes. These findings suggest that high-dose filgrastim may contribute to improvement of cytopenias and promote the recovery of immune cell populations (Table 5 and Table 6).

### 3.3. Survival Outcomes

The mortality rates among the cats included in the study were determined as 53.8% (7/13) in the LDF group, 42.9% (6/14) in the HDF group, 50% (6/12) in the iPPVO group, and 70% (7/10) in the ST group. Nevertheless, on an observational basis, the survival rate was higher in the HDF group (57.1%) compared with the other groups (Table 7).

## 4. Discussion

Feline panleukopenia is characterized by severe leukopenia, lymphopenia, and neutropenia resulting from the suppression of bone marrow precursor cells due to the tropism of FPV for rapidly dividing cells [4,12]. In the present study, the detection of widespread cytopenias across all groups at baseline suggests that the cases were evaluated during the acute and severe phase of the disease. The marked tropism of FPV for cells with high mitotic activity, particularly bone marrow precursor cells, intestinal crypt epithelial cells, and lymphoid tissues, constitutes the primary pathogenetic mechanism underlying the severe panleukopenia observed during the acute phase of the disease [4].

In the present study, high-dose filgrastim was associated with a more pronounced hematological response in FPV-infected cats, particularly in leukocyte parameters. The accelerated leukocyte recovery observed in our high-dose filgrastim group is consistent with previous veterinary studies reporting the efficacy of rHuG-CSF in cats, including faster neutrophil recovery and improved clinical outcomes [3,19,36,37,38,39,40]. This observation aligns with the known mechanism of filgrastim as a recombinant human granulocyte colony-stimulating factor (r-metHuG-CSF), which binds to neutrophil precursors in the bone marrow, promotes their differentiation and maturation, and enhances the release of mature neutrophils into the circulation [40]. These findings suggest that filgrastim can effectively counteract the acute leukopenia induced by FPV infection, supporting its role as a valuable adjunctive therapy in affected cats. However, it should be noted that while leukocyte parameters improved, erythrocyte variables appeared largely unaffected by filgrastim treatment, highlighting that its hematopoietic effects may be primarily limited to the granulocytic lineage.

In this study, the progression of clinical signs and changes in body temperature were evaluated in cats diagnosed with FPL under different treatment protocols. At baseline (day 0), the high prevalence of gastrointestinal and systemic clinical signs, including vomiting, diarrhea, anorexia, and lethargy, across all groups was consistent with the typical acute clinical presentation of FPL and in agreement with previous reports [3,24,41]. By day 7, a reduction in the frequency of clinical signs was observed in all groups; however, fewer cats in the filgrastim-treated groups (particularly the HDF group) and in the iPPVO group exhibited clinical signs such as vomiting, diarrhea, anorexia, and lethargy compared with the ST group. The complete resolution of vomiting in the HDF and iPPVO groups, in contrast to its persistence in 30% (3/10) of cases in the ST group, suggests that these treatment approaches may be more effective in controlling gastrointestinal manifestations. Dascalu et al. [3] state that consistent combined treatment approaches are necessary to manage the serious clinical manifestations caused by FPV. Similarly, the continued presence of diarrhea, anorexia, and lethargy at higher rates in the ST group indicates that standard supportive therapy alone may be insufficient to support clinical recovery. These findings suggest that filgrastim may indirectly contribute to the reduction in clinical symptoms through its stimulatory effects on the hematopoietic system.

Filgrastim administration (5 µg/kg) has been shown to support hematological recovery in FPV-infected cats by inducing significant increases in WBC, lymphocyte, and granulocyte counts between days 0 and 7 [24]. In the present study, marked and statistically significant increases in WBC, lymphocyte, monocyte, and granulocyte counts were observed on day 7 in the filgrastim-treated groups, particularly in the HDF group (20 µg/kg). These findings are consistent with previous reports indicating that filgrastim increases leukocyte counts in neutropenic cats and dogs [3,20,40,42]. However, it has also been reported that rHuG-CSF therapy does not result in a marked increase in leukocyte counts in some clinical cases of FPL [15]. Barrs et al. [11] reported that the response to filgrastim administration could vary depending on the dose and individual variability. For this reason, it was considered that the more pronounced dose-dependent response observed in the HDF group might be closely related to the administered dose of filgrastim.

On day 7 following treatment, the pronounced leukocytosis and granulocytosis observed in the HDF group are consistent with previous studies describing the granulocytic stimulatory effects of filgrastim on the bone marrow. Barrs et al. [11] reported that recombinant G-CSF administration in FPV cases could increase neutrophil counts, although the response was both dose-dependent and subject to individual variability. On the other hand, the literature has also reported that high-dose G-CSF administration can lead to excessive or irregular granulocyte responses, potentially increasing inflammatory burden [43,44]. The concurrent occurrence of granulocytosis and monocytosis in the HDF group suggests that, in some cases, the immune response may still be in an active inflammatory phase, aligning with the view that filgrastim primarily promotes cellular proliferation rather than immunoregulatory effects [43,44].

The iPPVO paraimmune activator has been reported to enhance the innate immune response by stimulating the release of interferons and various cytokines [32,45]. Weber et al. [32] reported that iPPVO activates both innate and adaptive immune responses through interferons and Th1-associated cytokines, without inducing excessive cellular proliferation. In the present study, a moderate increase in WBC and granulocyte counts was observed in the iPPVO group; however, this increase was not as pronounced as in the filgrastim-treated groups, particularly the HDF group. This finding may indicate that iPPVO exerts an indirect immunomodulatory effect rather than directly stimulating the bone marrow [32]. Furthermore, the results are consistent with the observations of De Mari et al. [46], suggesting that a balanced immune response in FPV cases may be more advantageous in terms of prognosis. Nevertheless, improvement of cytopenias in some cases within the iPPVO group does not entirely preclude the potential benefit of this agent as an adjunctive therapy.

In the present study, ANCOVA analyses revealed no statistically significant differences among the groups in erythrocyte parameters (RBC, Hgb, Hct) or platelet counts, supporting the notion that FPV infection primarily affects the leukocyte series during the acute phase [47,48]. In contrast, Dascalu et al. [3] reported significant decreases in RBC, Hgb, and Hct after treatment. Although not statistically significant in our study, numerical decreases in these parameters were observed in repeated within-group measurements, which is consistent with reports that filgrastim administration may occasionally be associated with side effects such as anemia [3].

Rice [19] reported 100% recovery following filgrastim treatment in FPV cases younger than three months. However, the survival rates observed in our study were considerably lower; particularly, the survival rate in cats aged 0–3 months was only 21.4%. In the filgrastim-treated group, the survival rate was 25% for cats under three months, whereas it reached 63.2% in cats aged three months and older. Similarly, Kızılkaya and Kara [39] reported survival rates of 50% in filgrastim-treated cats younger than three months and older than 12 months, and 70% in those aged 3–12 months. The differences between these studies may be attributed to the progressive nature of the disease or variations in disease severity at the time of presentation.

Although no statistically significant difference in survival rates was observed between the groups in this study, the HDF group exhibited a higher survival rate (57.1%) compared to the other groups. Felix et al. [38] reported that six out of seven cats survived following administration of filgrastim at a dose of 5 µg/kg. Kızılkaya and Kara [39] indicated that filgrastim treatment increased survival rates threefold. Rice [19] reported a 90% survival rate with filgrastim administration, attributing this high success rate to early diagnosis, aggressive symptomatic therapy, and the addition of filgrastim treatment. Therefore, the lack of a statistically significant translation of marked hematological improvement into survival may be related to the limited sample size. Indeed, the literature emphasizes that the efficacy of filgrastim can vary depending on the degree of neutropenia and the timing of treatment initiation [3]. Nevertheless, when mortality rates across the groups are examined, the lowest mortality was observed in the HDF group (42.86%), followed by the LDF (53.85%) and iPPVO (50%) groups. This finding suggests that high-dose filgrastim administration may positively influence prognosis, which is consistent with previously reported outcomes [19,39]. However, Uyurca and Haydardedeoğlu [24] stated that filgrastim application had no effect on mortality. Consequently, survival in FPV infection appears to be influenced not only by hematological recovery but also by supportive care, control of secondary infections, and the severity of disease at presentation [11,49].

In the literature, a decrease in leukocyte counts during FPV infection has been reported as a critical prognostic marker, with neutropenia showing a particularly strong association with mortality [4,10,50,51]. Similarly, granulocytopenia is regarded as an early and prominent indicator of bone marrow suppression [51]. Consistently, in the present study, median granulocyte counts on day 7 were significantly higher in surviving FPL-infected cats compared to those that succumbed to the infection. Kızılkaya and Kara [39] reported that WBC, Lymp, and Neu values were significantly higher after treatment, but this did not cause a statistically significant difference in prognosis. Similarly, Uyurca and Haydardedeoğlu [24] observed an increase in WBC, Lymp and granulocyte counts after filgrastim application, but concluded that this had no effect on mortality. This suggests that the evaluation of WBC, Lymp, and Neu values as survival markers should be interpreted with caution. The finding of higher granulocyte counts in surviving cats in the present study may indicate that hematological recovery progresses in parallel with clinical improvement. Therefore, when both studies are considered together, it can be said that changes in leukocyte and granulocyte values in FPV infection are important indicators reflecting the course of the disease; however, the relationship between these parameters and prognosis may vary depending on clinical and methodological factors.

This study has several limitations. The sample size was relatively small, and the number of cats receiving each treatment varied, leading to unequal group sizes. Hematological parameters were assessed only on days 0 and 7; viral load was not quantified, and long-term follow-up data were lacking. Supportive care, vaccination status, breed distribution and other clinical variables were not fully standardized, and the study was conducted at a single veterinary teaching hospital in Kayseri, Türkiye, which may limit external validity. Therefore, these findings should be interpreted as preliminary observational data.

## 5. Conclusions

In conclusion, this study suggests that high-dose filgrastim was associated with greater hematological improvement in cats with FPV infection compared with low-dose filgrastim, iPPVO, and standard treatment. Baseline-adjusted analyses indicated that treatment effects were mainly observed in leukocyte parameters, whereas erythrocyte variables were largely determined by baseline values and were not significantly influenced by treatment. These findings indicate that filgrastim may be a useful adjunctive therapy for supporting hematological recovery in FPV-infected cats. However, given the lack of a significant survival benefit, baseline imbalances, and the study’s analytical limitations, these results should be interpreted with caution. Further large, well-controlled randomized studies are needed to confirm these observations, clarify the clinical impact of filgrastim and iPPVO in FPV infection, and generalize the findings to other clinical settings.

## Figures and Tables

**Table 1:** Distribution of cats in the LDF, HDF, iPPVO, and ST groups according to age, sex, breed, and vaccination status, and intergroup comparisons.

	LDF (n = 13)	HDF (n = 14)	iPPVO (n = 12)	ST (n = 10)	*p* Value
Age (Month) *	12 (5.5–12)	5.5 (2.75–8.25)	5.50 (4–7.50)	12 (4.75–18)	*p* = 0.611
Gender					
*Male*	46.2 (6/13)	64.3 (9/14)	41.7 (5/12)	60 (6/10)	*p* = 0.656 χ^2^ = 1.809
*Female*	53.8 (7/13)	35.7 (5/14)	58.3 (7/12)	40 (4/10)	
Breed					
*British Shorthair*	53.8 (7/13)	28.6 (4/14)	33.3 (4/12)	10 (1/10)	*p* = 0.095 χ^2^ = 15.200
*Scottish Fold*	30.8 (4/13)	7.1 (1/14	8.3 (1/12)	30 (3/10)	
*Tabby cat*	-	64.3 (9/14)	50 (6/12)	60 (6/10)	
*Crossbreed*	15.4 (2/13)	-	-	-	
*Van Cat*	-	-	8.3 (1/12)	-	
Vaccination Status					
*Vaccinated*	7.7 (1/13)	50 (7/14)	8.3 (1/12)	40 (4/10)	*p* < 0.001 χ^2^ = 21.993
*Unvaccinated*	23.1 (3/13)	50 (7/14)	91.7 (11/12)	40 (4/10)	
*Unknown*	69.2 (9/13)	-	-	20 (2/10)	

Data were expressed as % (n/total), *: Median (1st quartile–3rd quartile).

**Table 2:** Comparison of clinical findings and body temperature values on days 0 and 7 according to treatment groups.

Clinical Findings	Days	LDF (n = 13)	HDF (n = 14)	iPPVO (n = 12)	ST (n = 10)
Vomiting	Day 0	100 (13/13)	100 (14/14)	91.67 (11/12)	90 (9/10)
	Day 7	7.69 (1/13)	0 (0/14)	0 (0/12)	30 (3/10)
Diarrhea	Day 0	76.92 (10/13)	92.86 (13/14)	83.33 (10/12)	100 (10/10)
	Day 7	15.39 (2/13)	14.29 (2/14)	16.66 (2/12)	50 (5/10)
Lack of appetite	Day 0	100 (13/13)	100 (14/14)	100 (12/12)	100 (10/10)
	Day 7	23.08 (3/13)	14.29 (2/14)	25 (3/12)	50 (5/10)
Lethargy	Day 0	100 (13/13)	100 (14/14)	100 (12/12)	100 (10/10)
	Day 7	38.46 (5/13)	21.43 (3/14)	33.33 (4/12)	70 (7/10)
Temp. (°C) *	Day 0	36.62 ± 1.77	36.72 ± 0.95	38.10 ± 2.51	37.53 ± 1.86
	Day 7	38.14 ± 0.69	38.02 ± 0.49	37.78 ± 0.62	37.25 ± 0.92
	*p* value	0.185	0.019	0.779	0.374

Data were expressed as % (n/total) and means ± standard deviation (*). LDF: Low-dose filgrastim, HDF: High-dose filgrastim, iPPVO: Inactive *Parapoxvirus ovis*, ST: Standard supportive treatment.

**Table 3:** Comparison of leucocyte parameters between LDF, HDF, iPPVO, and ST groups.

Parameters	Days	LDF (n = 13)	HDF (n = 14)	iPPVO (n = 12)	ST (n = 10)	ANCOVA *p* Value
WBC (10^9^/L)	Day 0	1.1 (0.2–3.8) ^A^	0.9 (0.2–1.9) ^A^	1.6 (0.3–3.2) ^AB^	3.2 (0.3–4.4) ^B^	-
	Day 7	2.8 (1.1–21.8) ^AC^	19.40 (1.0–79.5) ^B^	8.4 (2.4–34.4) ^AB^	4.8 (2.2–11.4) ^C^	0.001
	p*	0.017	0.001	0.006	0.110	
Lymp. (10^9^/L)	Day 0	0.4 (0.0–1.0) ^A^	0.5 (0.2–0.9) ^A^	0.4 (0.1–0.9) ^A^	1.2 (0.3–3.1) ^B^	-
	Day 7	0.8 (0.5–3.1) ^A^	2.2 (0.5–4.8) ^B^	1.2 (0.4–3.4) ^AB^	2.7 (0.8–4.2) ^B^	0.063
	p*	0.001	0.001	0.005	0.041	
Mono. (10^9^/L)	Day 0	0.1 (0.0–0.2)	0.1 (0.0–0.5)	0.2 (0.0–0.5)	0.2 (0.0–0.6)	-
	Day 7	0.3 (0.1–1.5) ^A^	1.0 (0.0–4.6) ^B^	0.5 (0.0–3.2) ^AB^	0.5 (0.1–0.6) ^A^	0.047
	p*	0.039	0.009	0.032	0.743	
Gran. (10^9^/L)	Day 0	0.3 (0.0–2.7)	0.4 (0.0–6.9)	0.9 (0.0–2.4)	0.5 (0.0–2.9)	-
	Day 7	2.0 (0.2–16.9) ^A^	14.8 (0.2–65.7) ^B^	6.4 (1.5–28.3) ^B^	0.9 (0.3–8.8) ^A^	<0.001
	p*	0.023	<0.001	0.008	0.201	
Eos (%)	Day 0	1.6 (0.3–10.5)	2.6 (0.1–25.2)	5.1 (0.1–13.1)	3.7 (2.1–5.5)	-
	Day 7	3.7 (0.6–17.0)	2.70 (0.5–5.8)	1.80 (0.1–6.2)	3.9 (1.0–7.7)	0.120
	p*	0.485	0.821	0.011	0.738	

Data are presented as median (min–max). ANCOVA performed for day 7 values with baseline (day 0) values included as covariates. *p* values indicate statistical significance between groups, whereas p* values indicate within-groups significance for repeated measures. Capital letters within the same row and column indicate similarities/differences between groups. LDF: Low-dose filgrastim, HDF: High-dose filgrastim, iPPVO: Inactive *Parapoxvirus ovis*, ST: Standard supportive treatment, WBC: white blood cells, Lymp: lymphocytes, Mono: monocytes, Gran: neutrophils.

**Table 4:** Comparison of erythrocyte and platelet parameters between LDF, HDF, iPPVO, and ST groups.

Parameters	Days	LDF (n = 13)	HDF (n = 14)	iPPVO (n = 12)	ST (n = 10)	ANCOVA *p* Value
RBC (10^12^/L)	Day 0	8.06 ± 1.07	7.95 ± 2.02	8.49 ± 2.52	9.04 ± 2.34	-
	Day 7	7.46 ± 1.73	8.08 ± 1.49	7.29 ± 1.84	8.60 ± 2.11	<0.001
	p*	0.058	0.168	0.114	0.528	
Hgb (g/dL)	Day 0	11.87 ± 2.21	11.94 ± 2.98	13.32 ± 3.33	12.72 ± 3.41	-
	Day 7	11.49 ± 3.34	11.48 ± 2.19	11.02 ± 2.29	12.22 ± 2.60	0.007
	p*	0.537	0.141	0.046	0.593	
Hct (%)	Day 0	33.69 ± 6.35	33.33 ± 8.35	35.66 ± 9.03	34.84 ± 9.93	-
	Day 7	32.36 ± 8.61	32.31 ± 6.84	30.38 ± 6.44	34.72 ± 7.24	0.009
	p*	0.341	0.231	0.141	0.962	
Plt (10^9^/L)	Day 0	332.5 (221.3–795.0)	201 (39–938)	206 (44–409)	197.5 (68–390)	-
	Day 7	239 (43–968)	244.5 (74–568)	180 (33–476)	271.5 (82–515)	0.52
	p*	0.835	0.847	0.325	0.209	

Data are presented as median (min–max) and means ± standard deviation. ANCOVA performed for day 7 values with baseline (day 0) values included as covariates. *p* values indicate statistical significance between groups, whereas p* values indicate within-groups significance for repeated measures. LDF: Low-dose filgrastim, HDF: High-dose filgrastim, iPPVO: Inactive *Parapoxvirus ovis*, ST: Standard supportive treatment, RBC: red blood cells, Hgb: haemoglobin, Hct: haematocrit, Plt: platelets.

**Table 5:** Effects of LDF, HDF, iPPVO, and ST administration on leucocyte parameters in cats.

Parameters	Blood Condition	Days	LDF (n = 13)	HDF (n = 14)	iPPVO (n = 12)	ST (n = 10)
WBC (10^9^/L)	Leukopenia (n)	Day 0	92.31 (12)	100 (14)	100 (12)	60 (6)
		Day 7	53.85 (7)	21.43 (3)	16.67 (2)	70 (7)
	In range (n)	Day 0	7.69 (1)	0 (0)	0 (0)	40 (4)
		Day 7	38.46 (5)	28.57 (4)	83.33 (10)	30 (3)
Lymp. (10^9^/L)	Lymphopenia (n)	Day 0	76.92 (10)	92.86 (13)	75 (9)	40 (4)
		Day 7	61.54 (8)	28.57 (4)	41.67 (5)	10 (1)
	In range (n)	Day 0	23.08 (3)	7.14 (1)	25 (3)	60 (6)
		Day 7	38.46 (5)	71.43 (10)	58.33 (7)	90 (9)
Mono. (10^9^/L)	Monocytopenia (n)	Day 0	23.08 (3)	21.43 (3)	33.33 (4)	30 (3)
		Day 7	7.69 (1)	7.14 (1)	8.33 (1)	0 (0)
	In range (n)	Day 0	76.92 (10)	78.57 (11)	66.67 (8)	70 (7)
		Day 7	76.92 (10)	64.29 (9)	66.67 (8)	100 (10)
Gran. (10^9^/L)	Granulocytopenia (n)	Day 0	92.31 (12)	78.57 (11)	75 (9)	60 (6)
		Day 7	46.15 (6)	21.43 (3)	16.67 (2)	80 (8)
	In range (n)	Day 0	7.69 (1)	21.43 (3)	25 (3)	40 (4)
		Day 7	46.15 (6)	14.29 (2)	66.67 (8)	20 (2)
Eos (%)	Eosinopenia (n)	Day 0	15.38 (2)	7.14 (1)	0 (0)	0 (0)
		Day 7	0 (0)	0 (0)	0 (0)	0 (0)
	In range (n)	Day 0	61.54 (8)	71.43 (10)	41.67 (5)	90 (9)
		Day 7	69.23 (9)	78.57 (11)	91.67 (11)	80 (8)

Data were expressed as % (n). LDF: Low-dose filgrastim, HDF: High-dose filgrastim, iPPVO: Inactive *Parapoxvirus ovis*, ST: Standard supportive treatment, WBC: white blood cells, Lymp: lymphocytes, Mono: monocytes, Gran: neutrophils, Eos: eosinophils.

**Table 6:** Effects of LDF, HDF, iPPVO, and ST administration on erythrocyte and platelet parameters in cats.

Parameters	Blood Condition	Days	LDF (n = 13)	HDF (n = 14)	iPPVO (n = 12)	ST (n = 10)
RBC (10^12^/L)	Anemia (n)	Day 0	46.15 (6)	35.71 (5)	50 (6)	30 (3)
		Day 7	46.15 (6)	42.86 (6)	66.67 (8)	30 (3)
	In range (n)	Day 0	53.85 (7)	64.29 (9)	41.67 (5)	70 (7)
		Day 7	53.85 (7)	57.14 (8)	33.33 (4)	70 (7)
Hgb (g/dL)	↓ Hgb (n)	Day 0	23.08 (3)	28.57 (4)	41.67 (5)	20 (2)
		Day 7	38.46 (5)	35.71 (5)	33.33 (4)	30 (3)
	In range (n)	Day 0	76.92 (10)	71.43 (10)	50 (6)	70 (7)
		Day 7	61.54 (8)	64.29 (9)	66.67 (8)	70 (7)
Hct (%)	↓ Hct (n)	Day 0	53.85 (7)	42.86 (6)	50 (6)	50 (5)
		Day 7	61.54 (8)	50 (7)	58.33 (7)	40 (4)
	In range (n)	Day 0	46.15 (6)	57.14 (8)	50 (6)	50 (5)
		Day 7	38.46 (5)	50 (7)	41.67 (5)	60 (6)
Plt (10^9^/L)	Thrombocytopenia (n)	Day 0	38.46 (5)	42.86 (6)	41.67 (5)	30 (3)
		Day 7	15.38 (2)	42.86 (6)	25 (3)	10 (1)
	In range (n)	Day 0	38.46 (5)	50 (7)	58.33 (7)	70 (7)
		Day 7	61.54 (8)	57.14 (8)	75 (9)	90 (9)

Data were expressed as % (n). ↓: Low, LDF: Low-dose filgrastim, HDF: High-dose filgrastim, iPPVO: Inactive *Parapoxvirus ovis*, ST: Standard supportive treatment, RBC: red blood cells, Hgb: haemoglobin, Hct: haematocrit, Plt: platelets.

**Table 7:** Numbers and percentages (%) of mortality and survival in cats according to treatment groups.

	LDF (n = 13)	HDF (n = 14)	iPPVO (n = 12)	ST (n = 10)	*p* Values
Died (n = 26)	53.8% (7/13)	42.9% (6/14)	50% (6/12)	70% (7/10)	*p* = 0.636 χ^2^ = 1.826
Survived (n = 23)	46.2% (6/13)	57.1% (8/14)	50% (6/12)	30% (3/10)	

LDF: Low-dose filgrastim, HDF: High-dose filgrastim, iPPVO: Inactive *Parapoxvirus ovis*, ST: Standard supportive treatment.

## Data Availability

The datasets generated and analyzed during the current study, including the raw hematological data and statistical analysis outputs, are available from the corresponding author upon reasonable request.

## Abbreviations

The following abbreviations are used in this manuscript:

FPL	Feline panleukopenia
FPV	Feline panleukopenia virus
iPPVO	inactivated *Parapoxvirus ovis*
HDF	High-dose filgrastim
LDF	Low-dose filgrastim
ST	Standard supportive treatment
rHuG-CSF	Recombinant human granulocyte colony-stimulating factor
PCR	Polymerase chain reaction
WBC	White blood cell
RBC	Red blood cell
Plt	Platelet
